# Efficacy and safety of three species of *Rhodiola* L. in patients with chronic obstructive pulmonary disease: A systematic review and meta-analysis

**DOI:** 10.3389/fphar.2023.1139239

**Published:** 2023-04-06

**Authors:** Haichuan Yu, Ting Lei, Xiaojie Su, Lu Zhang, Zhouzhou Feng, Mengya Dong, Zheyu Hou, Hong Guo, Jian Liu

**Affiliations:** ^1^ Clinical Medicine Department, First Medical College, Lanzhou University, Lanzhou, China; ^2^ Critical Care Department, The First Hospital of Lanzhou University, Lanzhou, China; ^3^ Secretary’s Office, Gansu Province Maternity and Child Health Hospital, Gansu Province Central Hospital, Lanzhou, China

**Keywords:** *Rhodiola* L., chronic obstructive pulmonary disease, safety, efficacy, systematic review

## Abstract

**Background:** Chronic obstructive pulmonary disease (COPD) is characterized by chronic hypoxia, inflammation, oxidative stress, and irreversible airflow limitations. *Rhodiola* L*.* is a genus of botanical drugs used in traditional medicine that may influence COPD.

**Objective:** A systematic review of the safety and efficacy of *Rhodiola* L. in patients with COPD.

**Material and methods:** We searched the PubMed, Embase, Cochrane Library, Web of Science, Scopus, China National Knowledge Infrastructure (CNKI), Chongqing VIP, Wanfang, and SinoMed databases. The search strategy used terms including “COPD” and “Rhodiola.” Two independent reviewers conducted the literature screening, data extraction, and risk of bias assessment, with a third reviewer involved to resolve disagreements. Statistical analysis was conducted in Review Manager (version 5.4.1), following the Cochrane Handbook.

**Results:** This review included nine studies, of which two focused on *Rhodiola crenulata* (Hook.f. and Thomson) H. Ohba (R. *crenulata*) and two on *Rhodiola kirilowii* (Regel) Maxim (R. *kirilowii*); the remaining five focused on *Rhodiola wallichiana* (Hook.) S.H.Fu (R. *wallichiana*). Compared with the placebo, patients who received *Rhodiola* L. presented no more adverse events (*p* = 0.65) but showed significant improvement in the percentage of forced expiratory volume in 1 s at prediction (FEV1%pred), forced expiratory volume in 1 s (FEV1), the ratio of forced expiratory volume in 1 s on forced vital capacity (FEV1/FVC), saturation of oxygen in arterial blood, partial pressure of oxygen in arterial blood (PaO_2_), partial pressure of carbon dioxide in arterial blood (PaCO_2_), systolic pulmonary arterial pressure, diastolic pulmonary arterial pressure, COPD assessment test, efficient rate, C-reactive protein, and N-terminal pro-B-type natriuretic peptide (all *p* < 0.01). Compared with ambroxol, R. *kirilowii* provided additional benefits to patients with COPD in FEV1%pred, FEV1, FEV1/FVC, PaO2, PaCO2, 8-iso-prostaglandin F2α, superoxide dismutase, glutathione, malondialdehyde, and total antioxidant capacity (all *p* < 0.01).

**Conclusion:** Among the *Rhodiola* L. genus, this review included R. *wallichiana*, R. *crenulata*, and R. *kirilowii*, which might be safe and effective in COPD. Although this study has several limitations, further RCTs are needed.

**Systematic Review Registration**: [https://www.crd.york.ac.uk/PROSPERO/ display_record.php?RecordID=302881], identifier [CRD42022361890].

## 1 Introduction

Chronic obstructive pulmonary disease (COPD) is the third-leading cause of mortality globally (GBD Chronic Respiratory Disease Collaborators, 2020). COPD is a chronic respiratory disease characterized by irreversible airflow limitations. It consists of complicated pathophysiological processes including hypoxia, oxidative stress, and sustained systematic or local inflammation (GOLD Report, 2023). Based on these changes, COPD leads to many typical manifestations including continuous dyspnea, cough, chronic fatigue, and impaired quality of life. The conventional treatment of COPD is inhaled bronchodilators or corticosteroids (GOLD Report, 2023). However, COPD is not just an acute local inflammatory disease; therefore, these treatments are insufficient and the control of deterioration by such methods is usually not satisfying in some patients.

*Rhodiola L.* is a genus of botanical drugs broadly used as adaptogens. It has a long history of use and has been applied in traditional medicine throughout Asia and Europe. It has also been recorded in the official pharmacopeia of several countries, including China and Russia, because of its widespread clinical application and its proven efficacy in clinical trials for a variety of diseases (Panossian et al., 2021). *Rhodiola L.* is widely distributed in the high altitudes of Asia and Europe and was generally used to relieve altitude sickness. In traditional Chinese medicine, *Rhodiola L.* is believed to tonify the Qi and benefit the lungs, while in modern medicine, *Rhodiola L.* reportedly alleviates dyspnea, chronic cough, and fatigue (Chinese Pharmacopoeia Commission, 2020), which are the major clinical manifestations of COPD (GOLD Report, 2023). Several bioactive substances have been separated from *Rhodiola L.*, including salidroside, tyrosol, and ethyl gallate (Han et al., 2016). These ingredients provide anti-inflammation, anti-overoxidation, and anti-depression effects, among others. (Li et al., 2017). Based on these mechanisms, several studies confirmed the efficacy of *Rhodiola L.* in many diseases. For instance, a comprehensive supplementation including *Rhodiola* L. reduced stress levels in healthy individuals (Noah et al., 2022)^,^ (Boyle et al., 2022). *Rhodiola* L. also improved explosive physical training performance in healthy participants (Williams et al., 2021). *Rhodiola* L. also improved depression symptoms and the quality of life of depressant patients (Gao et al., 2020)^,^ (Mao et al., 2015). In patients with respiratory diseases other than COPD, for instance, in obstructive sleep apnea, *Rhodiola* L. supplementation not only improved patient mental conditions but also elevated serum anti-oxidant concentrations (Yu et al., 2019).

However, despite the hypothesis that *Rhodiola* L. might have some benefits in patients with COPD, there remains no consensus on whether *Rhodiola* L. is efficient and safe in this population. One challenge is several species of *Rhodiola* L. are applied in similar medical use. For example, in China, extracts of *Rhodiola crenulata* (Hook.f. and Thomson) H. Ohba (R. *crenulata*), *Rhodiola wallichiana* (Hook.) S.H.Fu (R. *wallichiana*), and *Rhodiola kirilowii* (Regel) Maxim (R. *kirilowii*) are marketed (“*Rhodiola crenulata* (Hook.f. and Thomson) H. Ohba.,” 2023; “*Rhodiola kirilowii* (Regel) Maxim.,” 2023; “*Rhodiola wallichiana* (Hook.) S.H.Fu.,” 2023). Several randomized controlled trials (RCTs) on this topic reported different outcomes. For instance, one study on the efficacy of R. *crenulata* in COPD assessed patient lung function, symptom score, and physical endurance, while another study focused on inflammatory biomarkers (Chen et al., 2015; Chuang et al., 2015). Moreover, contrary results have been reported. For example, two studies reported that the addition of R. *wallichiana* significantly elevated the efficient rate of COPD treatment (Qv et al., 2014; Li et al., 2016), while Zhu et al. (2015) observed no significant difference between the *Rhodiola L.* and placebo groups. These findings make it difficult to summarize the information on this topic. Therefore, we conducted this systematic review and meta-analysis to make conclusions based on existing evidence from RCTs.

## 2 Methods

This systematic review was conducted following the Preferred Reporting Items for Systematic Reviews and Meta-Analyses (PRISMA) statement and preliminarily registered on the International Prospective Register of Systematic Reviews (PROSPERO, #CRD 42022361890) (Page et al., 2021; Yu et al., 2023). This study was designed and conducted following the guidance of the Cochrane Handbook for Systematic Reviews of Interventions (Higgins et al., 2022). Moreover, this report followed the “Consensus statement on the Phytochemical Characterisation of Medicinal Plant extracts” (ConPhyMP) guideline (Heinrich et al., 2022).

### 2.1 Literature search and screening

The PubMed, Cochrane Library, Web of Science, Scopus, Embase, ClinicalTrials.gov, China National Knowledge Infrastructure, Chongqing VIP, Wanfang, and SinoMed databases were searched for potential literature studies. Major terms used in the literature search were “Rhodiola” and “COPD.” The detailed search strategy and results are presented in Supplementary Table S1. Articles written in English or Chinese and published from the inception of databases to 30 September 2022 were included. Two reviewers independently screened the included literature, and a third experienced reviewer combined the two screening results by discussing them with the other reviewers.

### 2.2 Eligibility criteria for the literature screening

The eligibility criteria used in the screening process were 1) participants: adult patients with spirometry or physician-confirmed COPD; 2) intervention: drugs in which *Rhodiola* L. was the main ingredient, regardless of administration type; 3) comparators: placebo or conventional treatment (including monotherapy or combined bronchodilator or corticosteroid therapy); 4) outcomes: as the outcomes were inconsistent, we did not set limits on this domain and performed a meta-analysis of any indicators reported by at least two studies; and 5) study design: only RCTs.

### 2.3 Data extraction and quality assessment

Two reviewers independently extracted data from the finally retrieved articles using a pre-designed data form. These items included the following: 1) basic study information: first author, publication year, and PICOS information; 2) the risk of bias information defined in Cochrane Review Manager 5.4.1; and 3) the data on efficacy and safety outcomes. We did not target specific outcomes. Instead, we comprehensively gathered every outcome and meta-analyzed any outcome that was reported by at least two studies.

We assessed the quality of included studies using the Cochrane Risk of Bias (RoB) Tool (1.0) provided in RevMan 5.4. The RoB assessment contains seven domains: random sequence generation, allocation concealment, blinding of participants and personnel, blinding of outcome assessment, incomplete outcome, selective reporting, and other bias. Two different reviewers independently assessed all included studies, and a third experienced reviewer combined the two assessing results.

### 2.4 Data analysis

All data analysis was conducted in RevMan (version 5.4.1, The Cochrane Collaboration). We systematically reviewed and meta-analyzed two types of data (dichotomous and continuous). For dichotomous data, the risk ratio (RR) was calculated; for continuous data, the mean difference (MD) or standardized mean difference (SMD) was calculated. When the continuous data were tested using different methods and mechanisms or presented in different units, SMD was used; otherwise, the MD was used. Along with the pooling estimations, the 95% confidence interval (95%CI) was also. Heterogeneity was analyzed using the *I* ^*2*^ test. When *I* ^*2*^ > 50% and *p* < 0.05, a random-effects model (REM) was used; otherwise, a fixed-effects model (FEM) was used in the pooling process. For outcomes that were pooled by REM, we further conducted sensitivity analysis by omitting one study at a time. Although Egger et al. implied that the results of publication bias tests for outcomes with <10 included studies may be unreliable, we generated funnel plots for outcomes with >5 included studies (Egger et al., 1997).

## 3 Results

**FIGURE 1:**
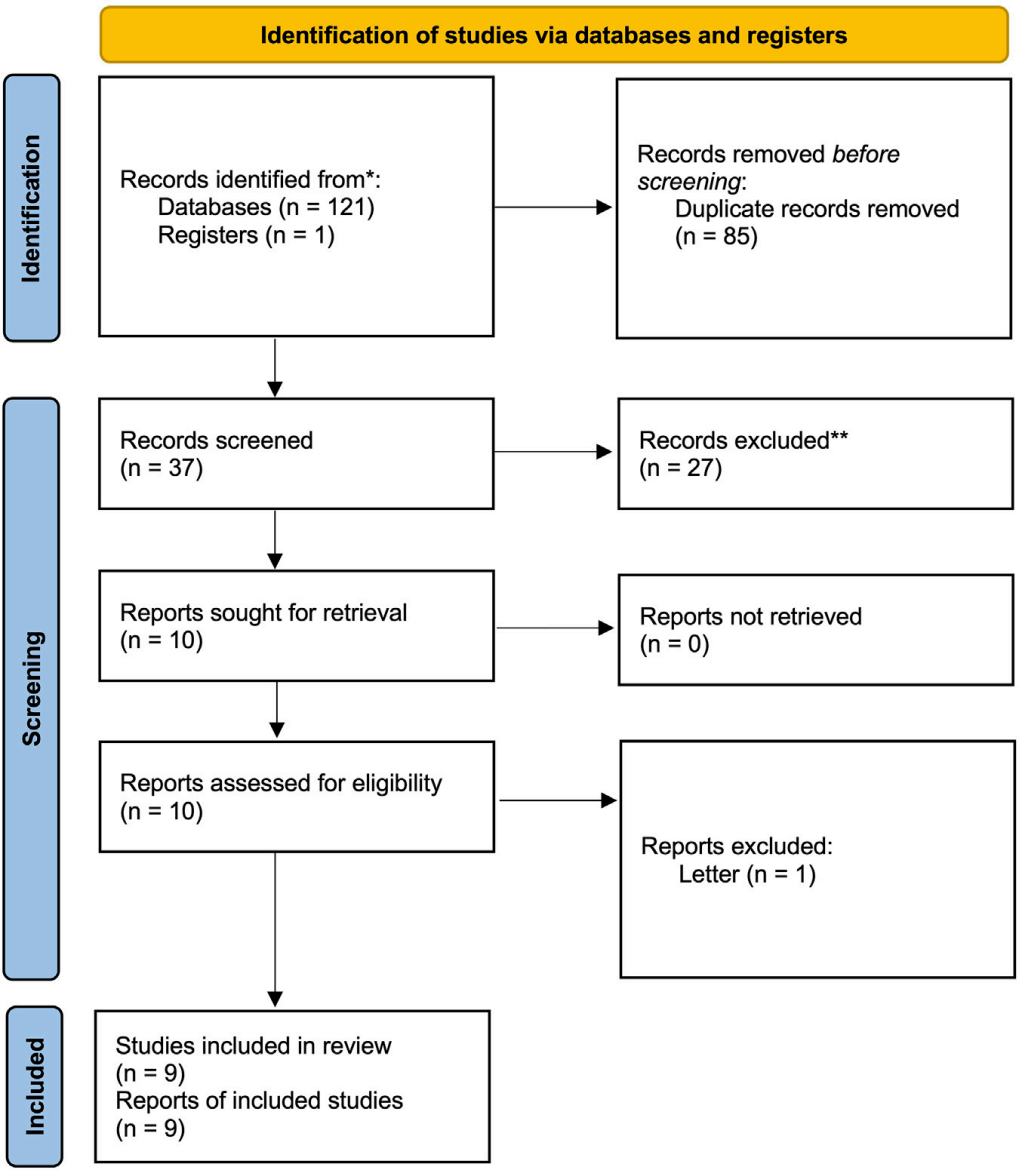
PRISMA 2020 checklist.

**FIGURE 2:**
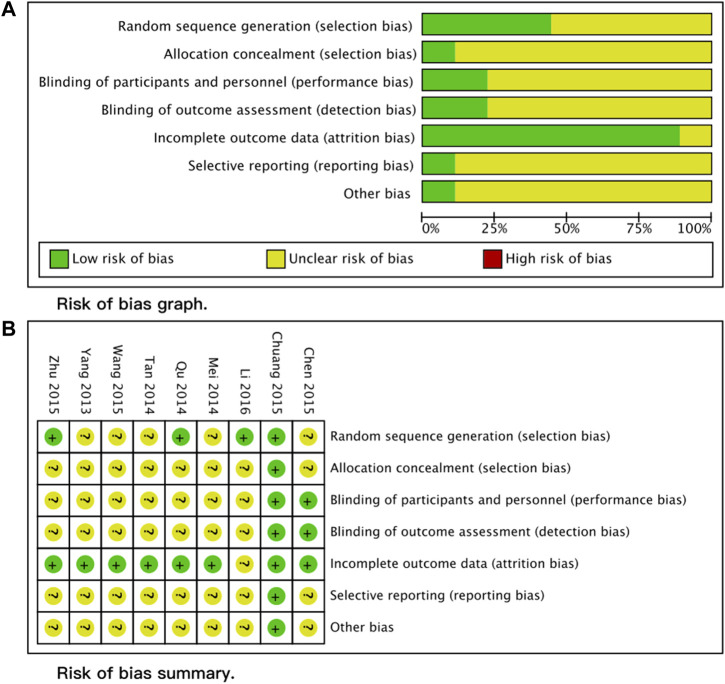
Risk of bias assessment **(A)** Risk of bias graph. **(B)** Risk of bias summary.

We identified 122 articles in the search process. After reading the titles and abstracts, we retrieved the full texts of 10 studies. One study was excluded after reading the full article because it was a letter, and no sufficient information could be extracted (Wang, 2010). Therefore, this study finally included nine studies (Wang, 2010; Yang et al., 2013; Mei et al., 2014; Qv et al., 2014; Tan, 2014; Chen et al., 2015; Chuang et al., 2015; Zhu et al., 2015; Li et al., 2016). The detailed screening process is presented in the PRISMA flow diagram (Figure 1). The overall quality of the included studies was medium, with the lack of detailed descriptions of randomization and masking the major reason impairing the quality of included studies. The detailed RoB assessment information is shown in Figure 2. Of all nine included articles, two were reported in English; the remaining seven were reported in Chinese, and all the studies were conducted in the Chinese population. Seven trials compared *Rhodiola* L. with placebo, while the other two trials compared *R*. *kirilowii* with ambroxol. Therefore, we performed separate analyses.

Two trials studied *R. crenulata*, two* R. kirilowii,* and five *R. wallichiana*. Detailed information on included studies is shown in Table 1. According to the ConPhyMP guidelines (Heinrich et al., 2022), all included studies focused on “type A” extracts (documented in national pharmacopeia with licensed application). Table 2 summarizes their preparations, dosage, routine, pharmaceutical manufacturer, etc.

**TABLE 1:** Basic characteristics of included studies.

Study ID	Sample size	Gender (F, M)	Age (years)	FEV1%pred	Basic treatment regimen^a^	Study direction
Chuang et al. (2015)	57 moderate to severe cases	1, 46	70.00, 8.80	61.83, 16.45	NR	MRE and AE
Chen et al. (2015)	57 moderate to severe cases	1, 46	49.53, 15.48	66.80, 1.66	NR	MRE
Li et al. (2016)	100 cases with PH	53, 47	54.72, 5.24	NR	Simvastatin, 20mg, qd, po, 2W	MRE, BB, and AE
Wang (2010)	60 cases with PH	26, 34	68.30, 2.40	NR	Fasudil, 30mg, qd, iv, 2W	MRE and AE
Zhu et al., 2015	96 cases with PH	30, 66	62.95, 5.23	NR	Atorvastatin, 20 mg, qd, po, 2W	MRE, BB, and AE
Mei et al. (2014)	60 cases with PH	25, 35	67.05, 3.47	NR	Fasudil, 30 mg, qd, iv, 2W	MRE and AE
Qv et al. (2014)	60 cases with PH	16, 44	78.00, 5.70	NR	NR	MRE and AE
Tan 2014	118 AE cases with PHD	40, 78	59.25, 11.31	35.30, 7.73	Ambroxol, 15 mg, bid, 4W**^b^**	MRE and BB
Yang et al. (2013)	126 AE cases with PHD	54, 72	58.67, 7.42	32.11, 5.65	Ambroxol, 30 mg, bid, 4W or NR^c^	MRE and BB

Abbreviations: F, female; M, male; FEV1%pred, percentage of forced expiratory volume in 1 s at prediction; PH, pulmonary hypertension; AE, acute exacerbation; PHD, pulmonary heart disease; NR, not reported; MRE, manifestation releasing efficacy; BB, blood biomarker; AE, adverse event.

Notes: Continuous data are presented as means and standard deviation.

^**a**^ “Basic treatment regimenˮ generally means extra treatments added to both the experimental and control groups.

^**b**^ *Tan (2014* *)* added ambroxol to the control group but not to the experimental group.

^c^ Yang et al., 2013 provided similar treatments as *Tan (2014* *)* but set an extra control group receiving neither ambroxol nor *Rhodiola*.

**TABLE 2:** Summary of intervention treatments.

Study ID	Preparation	Dosage	Frequency	Routine	Duration	Ingredient	Pharmaceutical manufacturer	Drug trade names
Chuang et al. (2015)	Capsule	250 mg	bid	po	12 weeks	*R. crenulata*	CSZ Phar Ltd.	紅景天濃縮膠囊
Chen et al. (2015)	Capsule	500 mg	bid	po	12 weeks	*R. crenulata*	CSZ Phar Ltd.	紅景天濃縮膠囊
Li et al. (2016)	Injection	10 ml	qd	iv	2 weeks	*R. wallichiana*	YS Phar Ltd.	大株红景天注射液
Wang (2010)	Injection	10 ml	qd	iv	2 weeks	*R. wallichiana*	YS Phar Ltd.	大株红景天注射液
Zhu et al. (2015)	Injection	10 ml	qd	iv	2 weeks	*R. wallichiana*	YS Phar Ltd.	大株红景天注射液
Mei et al. (2014)	Injection	10 ml	qd	iv	2 weeks	*R. wallichiana*	YS Phar Ltd.	大株红景天注射液
Qv et al. (2014)	Injection	10 ml	qd	iv	10 days	*R. wallichiana*	YS Phar Ltd.	大株红景天注射液
Tan (2014)	Capsule	0.2 g	tid	po	4 weeks	*R. kirilowii*	Sanpu Phar Ltd.	红景天胶囊
Yang et al. (2013)	Capsule	0.2 g	tid	po	4 weeks	*R. kirilowii*	Sanpu Phar Ltd.	红景天胶囊

Abbreviation: qd, quaque die, once daily; bid, bis in die, twice daily; tid, ter in die, three times daily; po, per os, oral; iv, intravenous. CSZ, Phar Ltd., Chuang Song Zong Pharmaceutical Company, Limited, Kaohsiung, Taiwan, China; YS, Phar Ltd., Yu Sheng Pharmaceutical Company, Limited, Tonghua, Jilin, China; Sanpu Phar Ltd., Sanpu Pharmaceutical Company, Limited, Xining, Qinghai, China; *R. crenulata*, *Rhodiola crenulata* (Hook.f. and Thomson) H. Ohba; *R. wallichiana*, *Rhodiola wallichiana* (Hook.) S.H.Fu**;** *R. kirilowii*, *Rhodiola kirilowii* (Regel) Maxim.

**FIGURE 3:**
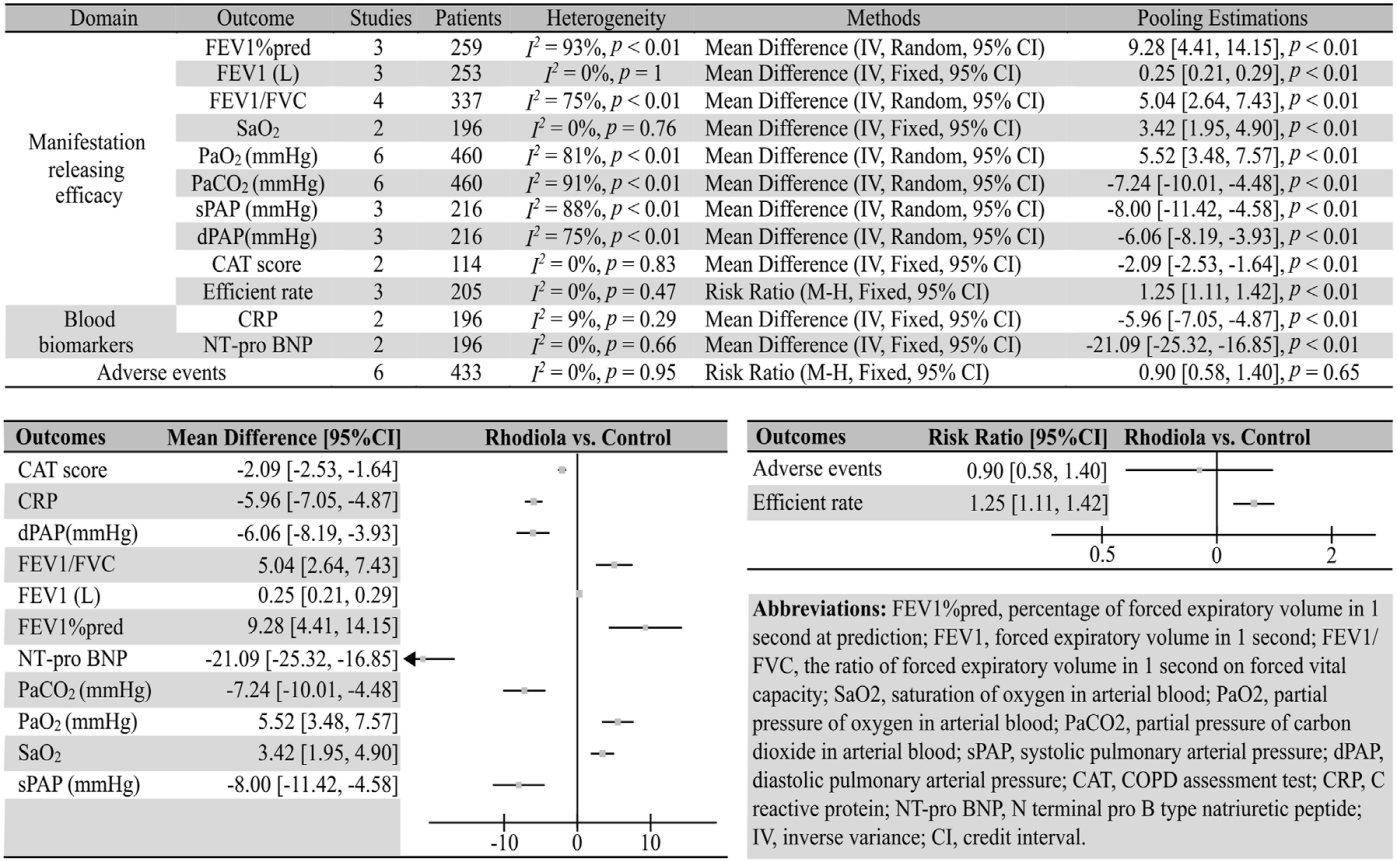
Summary of the meta-analysis results of *Rhodiola* vs. placebo in patients with COPD.

**FIGURE 4:**
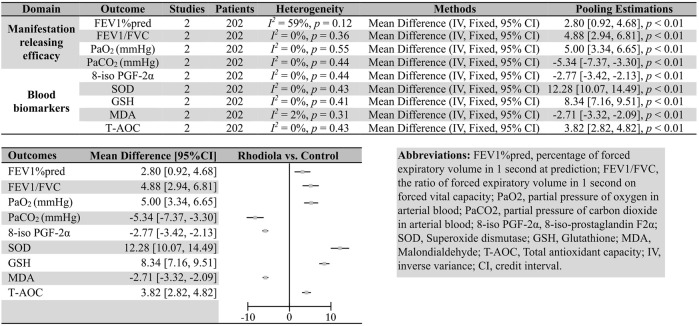
Summary of the meta-analysis results of *Rhodiola* vs. ambroxol in patients with COPD.

Two comparisons were made in included articles: *Rhodiola* L. vs. control and *Rhodiola* L. (only *R. kirilowii*) vs. ambroxol. The outcomes reported in these articles could be divided into four domains: manifestation-releasing efficacy, serum biomarkers, and safety concerns. We pooled the MD for every continuous outcome because all of them were tested and reported in the same manner. The meta-analysis results are summarized in Figures 3, 4. The related forest plots are shown in Supplementary Figures Series S1, S2. Sensitivity analysis was conducted by omitting the result of one study at a time for those pooling estimations for which *I* ^*2*^ > 50% and *p* < 0.05. All outcomes were robust to their heterogeneity. Subgroup analyses were conducted based on *Rhodiola* L. species.

### 3.1 Efficacy against COPD clinical manifestations

Compared with placebo, *Rhodiola* L. improved the FEV1%pred (three studies; 259 patients; *I* ^*2*^ = 93%, *p* < 0.01; REM; MD [95%CI] = 9.28 [4.41, 14.15], *p* < 0.01), FEV1 (three studies; 253 patients; *I* ^*2*^ = 0, *p* = 1; REM; MD [95%CI] = 0.25 [0.21, 0.29], *p* < 0.01), FEV1/FVC (four studies; 337 patients; *I* ^*2*^ = 75%, *p* < 0.01, REM; MD [95%CI] = 5.04 [2.64, 7.43], *p* < 0.01), SaO_2_ (two studies, 196 patients; *I* ^*2*^ = 0%, *p* = 0.76, FEM; MD [95%CI] = 3.42 [1.95, 4.90], *p* < 0.01), PaO_2_ (six studies, 460 patients; *I* ^*2*^ = 81%, *p* < 0.01, REM; MD [95%CI] = 5.52 [3.48, 7.57], *p* < 0.01), PaCO_2_ (six studies, 460 patients; *I* ^*2*^ = 91%, *p* < 0.01, REM; MD [95%CI] = −7.24 [−10.01, −4.48], *p* < 0.01), sPAP (three studies, 216 patients; *I* ^*2*^ = 88%, *p* < 0.01, REM; MD [95%CI] = −8.00 [−11.42, −4.58], *p* < 0.01), dPAP (three studies, 216 patients; *I* ^*2*^ = 75%, *p* < 0.01, REM; MD [95%CI] = −6.06 [−8.19, −3.93], *p* < 0.01), CAT score (two studies, 114 patients; *I* ^*2*^ = 0%, *p* = 0.83, FEM; MD [95%CI] = −5.96 [−7.05, −4.87], *p* < 0.01), and efficiency rate (three studies, 205 patients; *I* ^*2*^ = 0%, *p* = 0.47, FEM; RR [95%CI] = 1.25 [1.11, 1.42], *p* < 0.01).

Compared with ambroxol, *R*. *kirilowii* also showed superiority in several domains: FEV1%pred (two studies, 202 patients; *I* ^*2*^ = 59%, *p* = 0.12, FEM; MD [95%CI] = 2.80 [0.92, 4.68], *p* < 0.01), FEV1/FVC (two studies, 202 patients; *I* ^*2*^ = 0%, *p* = 0.36, FEM; MD [95%CI] = 4.88 [2.94, 6.81], *p* < 0.01), PaO2 (two studies, 202 patients; *I* ^*2*^ = 0%, *p* = 0.55, FEM; MD [95%CI] = 5.00 [3.34, 6.65], *p* < 0.01), and PaCO2 (two studies, 202 patients; *I* ^*2*^ = 0%, *p* = 0.44, FEM; MD [95%CI] = −5.34 [−7.37, −3.30], *p* < 0.01).

### 3.2 Efficacy in patient blood biomarkers

Compared with placebo or ambroxol, patients administered *Rhodiola* L. showed lower levels of blood biomarkers, CRP (vs. placebo; two studies, 196 patients; *I* ^*2*^ = 9%, *p* = 0.29, FEM; MD [95%CI] = -5.96 [-7.05, -4.87], *p* < 0.01), NT-pro BNP (vs. placebo; two studies, 196 patients; *I* ^*2*^ = 0%, *p* = 0.66, FEM; MD [95%CI] = −21.09 [−25.32, -16.85], *p* < 0.01), 8-iso PGF-2α (vs. ambroxol; two studies, 202 patients; *I* ^*2*^ = 0%, *p* = 0.44, FEM; MD [95%CI] = −2.77 [−3.42, −2.13], *p* < 0.01), SOD (vs. ambroxol; two studies, 202 patients; *I* ^*2*^ = 0%, *p* = 0.43, FEM; MD [95%CI] = 12.28 [10.07, 14.49], *p* < 0.01), GSH (vs. ambroxol; two studies, 202 patients; *I* ^*2*^ = 0%, *p* = 0.41, FEM; MD [95%CI] = 8.34 [7.16, 9.51], *p* < 0.01), MDA (vs. ambroxol; two studies, 202 patients; *I* ^*2*^ = 2%, *p* = 0.31, FEM; MD [95%CI] = −2.71 [−3.32, −2.09], *p* < 0.01), and T-AOC (vs. ambroxol; two studies, 202 patients; *I* ^*2*^ = 0%, *p* = 0.43, FEM; MD [95%CI] = 3.82 [2.82, 4.82], *p* < 0.01).

### 3.3 Safety of *Rhodiola* L. in patients with COPD

Six studies comparing *Rhodiola* L. and placebo reported that the *Rhodiola* L. groups did not show more adverse events (373 patients, *I* ^*2*^ = 0%, *p* = 0.65, FEM; RR [95%CI] = 0.90 [0.58, 1.40], *p* = 0.65).

### 3.4 Subgroup analysis

Depending on the *Rhodiola* L. species, the outcomes were divided into four categories: 1) only *R. crenulata* (vs. placebo: CAT score); 2) only *R. wallichiana* (vs. placebo: sPAP, dPAP, and SaO2); 3) *R. crenulata* or *R. kirilowii* with *R. wallichiana* (vs. placebo: FEV1%pred, FEV1, FEV1/FVC, PaO_2_, and PaCO_2_); and 4) only *R. kirilowii* (vs. ambroxol: all outcomes)*.* In category three, we conducted subgroup analyses to explore differences between *Rhodiola* L. species. However, results were inconsistent due to the limited number of included studies (see forest plots in the Supplementary Material).

### 3.5 Tests of publication bias

The analysis of PaO_2_, PaCO_2_, and adverse events included six studies, for which we plotted funnel plots. These plots are all symmetrical, suggesting a lack of significant publication bias (see Supplementary Figure Series).

## 4 Discussion

This systematic review and meta-analysis included nine studies and 734 patients. The results showed that *R. wallichiana*, *R. crenulata,* and *R. kirilowii* might be safe and promising in relieving the clinical manifestations, pulmonary circulation, inflammation, and oxidative stress of COPD. However, due to the lack of included studies, it might be too early to draw any clinically practical conclusions.

We found that *Rhodiola* L. may improve FEV1%pred, FEV1, FEV1/FVC, SaO_2_, PaO_2_, PaCO_2_, and CAT score, and efficient rate in COPD, indicating that *Rhodiola* L. might be beneficial to respiratory function and clinical manifestations in patients with COPD. Previous studies showed that *Rhodiola* L. might enhance exercise performance and pulmonary ventilation (Kelly, 2001; Zhang et al., 2009). However, the mechanisms of such effects remain unclear. The administration of *Rhodiola* L. improved adenosine triphosphate (ATP) turnover to increase muscle power (Abidov et al., 2003). The enhancement of the respiratory muscles after using *Rhodiola* L. might explain this slight recovery of respiratory function. The results of our subgroup analysis were based on limited evidence and are highly inconsistent; therefore, we cannot draw conclusions.

Our results showed that *Rhodiola* L. may improve sPAP, dPAP, and NT-pro BNP in COPD, indicating that *Rhodiola* L. might attenuate pulmonary hypertension (PH) in patients with COPD. Previous former studies confirmed the anti-PH effects *Rhodiola* L. and explored the mechanisms of these effects. Hypoxia is a key point in the occurrence and development of PH; therefore, in patients with COPD patients, hypoxia might also be a core mechanism for the development of PH (Seeger et al., 2013). *Rhodiola* L. inhibited the thickening of pulmonary vessel walls and maintained the vessel diameter in a PH rat model (Nan et al., 2018). The results of the present study also found that *Rhodiola* L. also inhibits the expression of several hypoxia-induced proteins, such as proliferating cell nuclear antigen (PCNA), cyclin-dependent kinase 4 (CDK4), and cyclin D1 in pulmonary tissue. *Rhodiola* L. also inhibited the proliferation of rat pulmonary artery smooth muscle cells (Nan et al., 2022; Zhang et al., 2022).

We found that *Rhodiola* L. may improve CRP, 8-iso PGF-2α, SOD, GSH, MDA, and T-AOC in COPD, indicating its potential anti-inflammatory and antioxidant effects in COPD. Previous studies demonstrated the anti-inflammatory potency of *Rhodiola* L. in many diseases including cancer, diabetes, and cardiovascular disease (Pu et al., 2020). This potency might be explained by its multiple metabolites’ inhibition of different inflammatory signaling pathways, including nuclear factor-*κ*-gene binding (NF- *κ*B), mitogen-activated protein kinase (MAPK), Janus kinase 2/signal transducer and activator of transcription 3 (JAK2/STAT3), and phosphoinositide 3-kinases/Akt (PI3K/Akt) (Chang et al., 2015; Je et al., 2015; Qi et al., 2016). Moreover, *Rhodiola* L. also demonstrated strong antioxidant activity (Sun et al., 2020), which can suppress the production of reactive oxygen species (ROS) and increase the activity of antioxidant agents such as superoxide dismutase (SOD) and glutathione peroxidase (GSH-Px) (Li et al., 2012; Xing et al., 2014).

This study is the first systematic review and meta-analysis to assess the efficacy and safety of *Rhodiola* L. in patients with COPD. It included only RCTs and combined mixed evidence to obtain an overall impression of *Rhodiola* L. in COPD. The study also has some limitations: 1) Although only RCTs were included, the Risk of Bias assessment of the included studies identified some “unclear risks”, which could weaken the strength of the evidence; 2) only nine studies with 734 patients were included; this small number of included trials and sample size limits the strength of the conclusion; 3) although publication bias was tested, the results remain doubtful because of the limited number of included studies. Well-designed, large-scale RCTs are still required; 4) all included studies analyzed the efficacy and safety of *Rhodiola* L. extract, without considering the specific substances contained in *Rhodiola* L. such as rosavin or salidroside; 5) this study included only the literature in Chinese and English; however, *Rhodiola* L. is used in many countries in Asia and Europe. Therefore, the possibility of articles related to this topic being published in other languages cannot be excluded. Therefore, language bias is possible. 6) *Rhodiola* L. is a large genus with many different species, while this study included only *R. wallichiana*, *R. kirilowii*, and *R. crenulata*; hence, the results can only be applied to these two species and cannot be generalized to other species.

This study analyzed several *Rhodiola* L. extracts currently available on the market. Limited by the number and quality of included studies, the inferences drawn are weak but are sufficient to raise awareness of these drugs by clinical practitioners. The safety and promising efficacy of *Rhodiola* L. extracts shown in this study indicated the need for more studies on this topic. As the current RCTs are generally not well designed, conducted, or reported, more effort must be put into these aspects in the future.

## 5 Conclusion

This study included *Rhodiola wallichiana* (Hook.) S.H.Fu, *Rhodiola kirilowii* (Regel) Maxim, and *Rhodiola crenulata* (Hook. f. and Thomson) H. Ohba, which might be safe and effective in COPD. They might be able to improve respiratory function, clinical manifestation, pulmonary circulation, inflammation, and oxidative stress. Limited by the quantity and quality of included studies, the results of this study are preliminary; therefore, well-designed, large-scale, randomized, controlled trials are required.

## Data Availability

The original contributions presented in the study are included in the article/Supplementary Material; further inquiries can be directed to the corresponding author.
