# Supplementary material for: Efficacy and safety of three species of Rhodiola L. in patients with chronic obstructive pulmonary disease: A systematic review and meta-analysis
Source: Front Pharmacol. 2023 Apr 6;14:1139239. doi: 10.3389/fphar.2023.1139239 (PMC10117638; doi:10.3389/fphar.2023.1139239)
Supplement: Supplementary file 2 [file DataSheet1.docx]

Supplementary Material

**Efficacy and Safety of *Rhodiola* L. in Patients with Chronic Obstructive Pulmonary Disease: A Systematic Review and Meta-analysis.**

**Haichuan Yu^1,2^, Xiaojie Su^1,2^, Ting Lei^1,2^, Lu Zhang^1,2^, Zhouzhou Feng^1,2^, Hong Guo^1,2^, Jian Liu^1,3,*^.**

^1^Clinical Medicine department, First Medical College, Lanzhou University, Chengguan District, Lanzhou City, Gansu Province, the People's Republic of China.

^2^Critical care department, the first hospital of Lanzhou University, Chengguan District, Lanzhou City, Gansu Province, the People's Republic of China.

^3^Critical care department, Gansu Provincial Maternal and Child Health Hospital, Chengguan District, Lanzhou City, Gansu Province, the People's Republic of China.

*** Correspondence:**Prof. Jian Liu

Phone number: +86 13609354197

Address: 222 Tianshui South Road, Chengguan District, Lanzhou City, Gansu Province, the People's Republic of China.

Email address: medecinliu@sina.com

**Supplementary Table 1 Search strategy**

| **Pubmed** | | |
| --- | --- | --- |
| No. | Query | Results |
| #5 | (#1 OR #3) AND (#2 OR #4) | 3 |
| #4 | "Rhodiola"[Title/Abstract] OR "Rhodiola crenulata"[Title/Abstract] OR "Rhodiola rosea"[Title/Abstract] OR "Roseroot"[Title/Abstract] OR "Roseroots"[Title/Abstract] | 1,073 |
| #3 | "Pulmonary Disease, Chronic Obstructive"[Title/Abstract] OR "chronic obstructive lung disease"[Title/Abstract] OR "Airflow Obstruction, Chronic"[Title/Abstract] OR "Airflow Obstructions, Chronic"[Title/Abstract] OR "Chronic Airflow Obstruction"[Title/Abstract] OR "Chronic Airflow Obstructions"[Title/Abstract] OR "chronic airway obstruction"[Title/Abstract] OR "Chronic Obstructive Airway Disease"[Title/Abstract] OR "Chronic Obstructive Pulmonary Disease"[Title/Abstract] OR "Chronic Obstructive Pulmonary Diseases"[Title/Abstract] OR "chronic obstructive bronchopulmonary disease"[Title/Abstract] OR "chronic obstructive lung disorder"[Title/Abstract] OR "chronic obstructive pulmonary disorder"[Title/Abstract] OR "chronic obstructive respiratory disease"[Title/Abstract] OR "chronic pulmonary obstructive disease"[Title/Abstract] OR "chronic pulmonary obstructive disorder"[Title/Abstract] OR "COAD"[Title/Abstract] OR "COPD"[Title/Abstract] OR "lung chronic obstructive disease"[Title/Abstract] OR "lung disease, chronic obstructive"[Title/Abstract] OR "obstructive chronic lung disease"[Title/Abstract] OR "obstructive chronic pulmonary disease"[Title/Abstract] OR "obstructive lung disease, chronic"[Title/Abstract] OR "pulmonary disorder, chronic obstructive"[Title/Abstract] | 80,918 |
| #2 | "Rhodiola"[Mesh] | 661 |
| #1 | "Pulmonary Disease, Chronic Obstructive"[Mesh] | 64,688 |
| **Embase** | | |
| No. | Query | Results |
| #5 | (#1 OR #3) AND (#2 OR #4) | 4 |
| #4 | 'rhodiola'/exp | 1,085 |
| #3 | 'chronic obstructive lung disease'/exp | 164,759 |
| #2 | 'rhodiola':ti,ab,kw OR 'rhodiola crenulata':ti,ab,kw OR 'rhodiola rosea':ti,ab,kw OR 'roseroot':ti,ab,kw OR 'roseroots':ti,ab,kw | 1374 |
| #1 | 'pulmonary disease, chronic obstructive':ti,ab,kw OR 'chronic obstructive lung disease':ti,ab,kw OR 'airflow obstruction, chronic':ti,ab,kw OR 'airflow obstructions, chronic':ti,ab,kw OR 'chronic airflow obstruction':ti,ab,kw OR 'chronic airflow obstructions':ti,ab,kw OR 'chronic airway obstruction':ti,ab,kw OR 'chronic obstructive airway disease':ti,ab,kw OR 'chronic obstructive pulmonary disease':ti,ab,kw OR 'chronic obstructive pulmonary diseases':ti,ab,kw OR 'chronic obstructive bronchopulmonary disease':ti,ab,kw OR 'chronic obstructive lung disorder':ti,ab,kw OR 'chronic obstructive pulmonary disorder':ti,ab,kw OR 'chronic obstructive respiratory disease':ti,ab,kw OR 'chronic pulmonary obstructive disease':ti,ab,kw OR 'chronic pulmonary obstructive disorder':ti,ab,kw OR 'coad':ti,ab,kw OR 'copd':ti,ab,kw OR 'lung chronic obstructive disease':ti,ab,kw OR 'lung disease, chronic obstructive':ti,ab,kw OR 'obstructive chronic lung disease':ti,ab,kw OR 'obstructive chronic pulmonary disease':ti,ab,kw OR 'obstructive lung disease, chronic':ti,ab,kw OR 'pulmonary disorder, chronic obstructive':ti,ab,kw | 140,680 |
| **Cochrane Library** | | |
| No. | Query | Results |
| #5 | (#1 OR #3) AND (#2 OR #4) | 5 |
| #4 | MeSH descriptor: [Rhodiola] explode all trees | 45 |
| #3 | MeSH descriptor: [Pulmonary Disease, Chronic Obstructive] explode all trees | 6,386 |
| #2 | (‘Rhodiola’ OR ‘Rhodiola crenulata’ OR ‘Rhodiola rosea’ OR ‘Roseroot’ OR ‘Roseroots’) :ti,ab,kw | 114 |
| #1 | ('Pulmonary Disease, Chronic Obstructive' OR 'chronic obstructive lung disease' OR 'Airflow Obstruction, Chronic' OR 'Airflow Obstructions, Chronic' OR 'Chronic Airflow Obstruction' OR ‘Chronic Airflow Obstructions’ OR ‘chronic airway obstruction’ OR ‘Chronic Obstructive Airway Disease’ OR ‘Chronic Obstructive Pulmonary Disease’ OR ‘Chronic Obstructive Pulmonary Diseases’ OR ‘chronic obstructive bronchopulmonary disease’ OR ‘chronic obstructive lung disorder’ OR ‘chronic obstructive pulmonary disorder’ OR ‘chronic obstructive respiratory disease’ OR ‘chronic pulmonary obstructive disease’ OR ‘chronic pulmonary obstructive disorder’ OR ‘COAD’ OR ‘COPD’ OR ‘lung chronic obstructive disease’ OR ‘lung disease, chronic obstructive’ OR ‘obstructive chronic lung disease’ OR ‘obstructive chronic pulmonary disease’ OR ‘obstructive lung disease, chronic’ OR ‘pulmonary disorder, chronic obstructive’):ti,ab,kw | 23,703 |
| **Web of Science** | | |
| No. | Query | Results |
| #1 | (TI=((Pulmonary Disease, Chronic Obstructive) OR (chronic obstructive lung disease) OR (Airflow Obstruction, Chronic) OR (Airflow Obstructions, Chronic) OR (Chronic Airflow Obstruction) OR (Chronic Airflow Obstructions) OR (chronic airway obstruction) OR (Chronic Obstructive Airway Disease) OR (Chronic Obstructive Pulmonary Disease) OR (Chronic Obstructive Pulmonary Diseases) OR (chronic obstructive bronchopulmonary disease) OR (chronic obstructive lung disorder) OR (chronic obstructive pulmonary disorder) OR (chronic obstructive respiratory disease) OR (chronic pulmonary obstructive disease) OR (chronic pulmonary obstructive disorder) OR (COAD) OR (COPD) OR (lung chronic obstructive disease) OR (lung disease, chronic obstructive) OR (obstructive chronic lung disease) OR (obstructive chronic pulmonary disease) OR (obstructive lung disease, chronic) OR (pulmonary disorder, chronic obstructive)) OR AK=((Pulmonary Disease, Chronic Obstructive) OR (chronic obstructive lung disease) OR (Airflow Obstruction, Chronic) OR (Airflow Obstructions, Chronic) OR (Chronic Airflow Obstruction) OR (Chronic Airflow Obstructions) OR (chronic airway obstruction) OR (Chronic Obstructive Airway Disease) OR (Chronic Obstructive Pulmonary Disease) OR (Chronic Obstructive Pulmonary Diseases) OR (chronic obstructive bronchopulmonary disease) OR (chronic obstructive lung disorder) OR (chronic obstructive pulmonary disorder) OR (chronic obstructive respiratory disease) OR (chronic pulmonary obstructive disease) OR (chronic pulmonary obstructive disorder) OR (COAD) OR (COPD) OR (lung chronic obstructive disease) OR (lung disease, chronic obstructive) OR (obstructive chronic lung disease) OR (obstructive chronic pulmonary disease) OR (obstructive lung disease, chronic) OR (pulmonary disorder, chronic obstructive)) OR AB=((Pulmonary Disease, Chronic Obstructive) OR (chronic obstructive lung disease) OR (Airflow Obstruction, Chronic) OR (Airflow Obstructions, Chronic) OR (Chronic Airflow Obstruction) OR (Chronic Airflow Obstructions) OR (chronic airway obstruction) OR (Chronic Obstructive Airway Disease) OR (Chronic Obstructive Pulmonary Disease) OR (Chronic Obstructive Pulmonary Diseases) OR (chronic obstructive bronchopulmonary disease) OR (chronic obstructive lung disorder) OR (chronic obstructive pulmonary disorder) OR (chronic obstructive respiratory disease) OR (chronic pulmonary obstructive disease) OR (chronic pulmonary obstructive disorder) OR (COAD) OR (COPD) OR (lung chronic obstructive disease) OR (lung disease, chronic obstructive) OR (obstructive chronic lung disease) OR (obstructive chronic pulmonary disease) OR (obstructive lung disease, chronic) OR (pulmonary disorder, chronic obstructive))) AND (TI=((Rhodiola) OR (Rhodiola crenulata) OR (Rhodiola rosea) OR (Roseroot) OR (Roseroots)) OR AK=((Rhodiola) OR (Rhodiola crenulata) OR (Rhodiola rosea) OR (Roseroot) OR (Roseroots)) OR AB=((Rhodiola) OR (Rhodiola crenulata) OR (Rhodiola rosea) OR (Roseroot) OR (Roseroots))) | 3 |
| **ClinicalTrials.gov** | | |
| No. | Query | Results |
| #1 | rhodiola \| COPD | NCT02242461 |
| **CNKI** | | |
| No. | Query | Results |
| #1 | (篇关摘=COPD + 慢阻肺 + 慢性阻塞性肺疾病) AND (篇关摘=红景天 + 景天 + rhodiola) | 25 |
| **Wan Fang** | | |
| No. | Query | Results |
| #1 | 题名或关键词:(COPD + 慢阻肺 + 慢性阻塞性肺疾病) and 题名或关键词:(红景天 + 景天 + rhodiola) | 32 |
| **Chongqing VIP** | | |
| No. | Query | Results |
| #1 | (((题名或关键词=COPD OR 题名或关键词=慢阻肺) OR 题名或关键词=慢性阻塞性肺疾病) AND ((题名或关键词=红景天 OR 题名或关键词=景天) OR 题名或关键词=rhodiola)) | 18 |
| **SinoMed** | | |
| No. | Query | Results |
| #1 | ( "COPD"[全部字段:智能] OR "慢阻肺"[全部字段:智能] OR "慢性阻塞性肺疾病"[全部字段:智能]) AND( "红景天"[全部字段:智能] OR "景天"[全部字段:智能] OR "rhodiola"[全部字段:智能]) | 27 |
| Scopus | | |
| No. | Query | Results |
| #1 | TITLE-ABS-KEY ( "Pulmonary Disease, Chronic Obstructive" OR "chronic obstructive lung disease" OR "Airflow Obstruction, Chronic" OR "Airflow Obstructions, Chronic" OR "Chronic Airflow Obstruction" OR "Chronic Airflow Obstructions" OR "chronic airway obstruction" OR "Chronic Obstructive Airway Disease" OR "Chronic Obstructive Pulmonary Disease" OR "Chronic Obstructive Pulmonary Diseases" OR "chronic obstructive bronchopulmonary disease" OR "chronic obstructive lung disorder" OR "chronic obstructive pulmonary disorder" OR "chronic obstructive respiratory disease" OR "chronic pulmonary obstructive disease" OR "chronic pulmonary obstructive disorder" OR "COAD" OR "COPD" OR "lung chronic obstructive disease" OR "lung disease, chronic obstructive" OR "obstructive chronic lung disease" OR "obstructive chronic pulmonary disease" OR "obstructive lung disease, chronic" OR "pulmonary disorder, chronic obstructive" ) AND TITLE-ABS-KEY ( "Rhodiola" OR "Rhodiola crenulata" OR "Rhodiola rosea" OR "Roseroot" OR "Roseroots" ) | 4 |

**Supplementary Figure Series 1 Forest plots (Rhodiola vs. Placebo)**

**
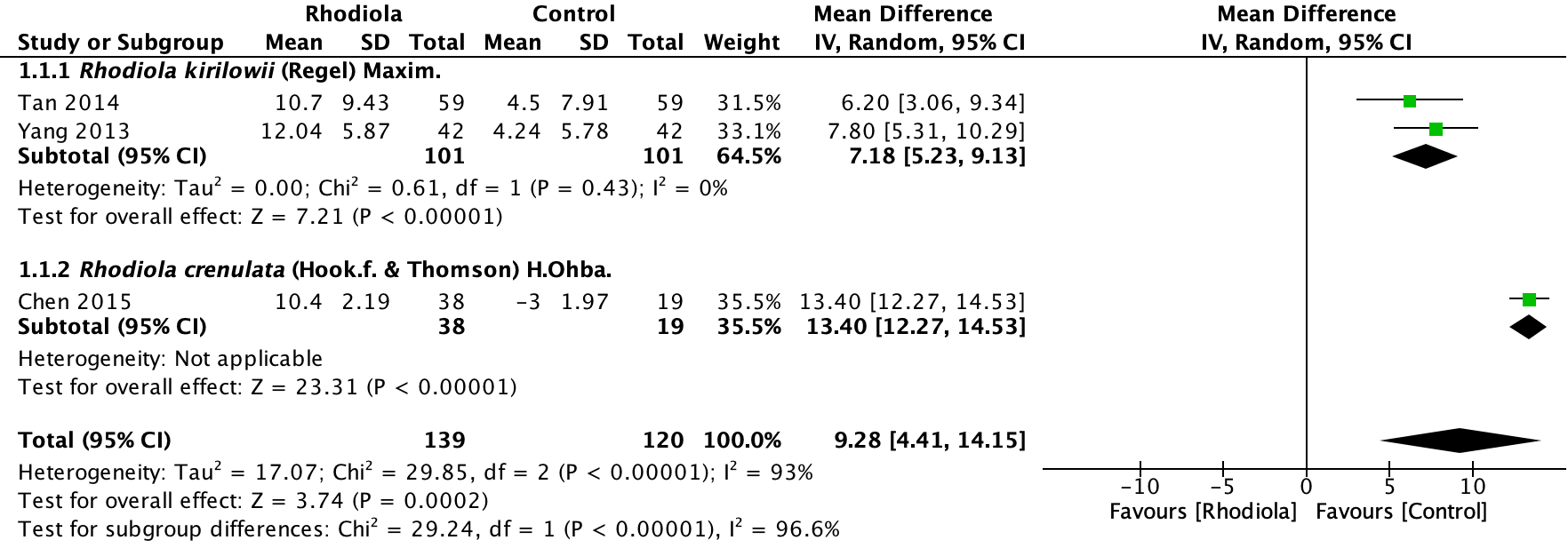
**

**Figure 1.1 FEV1%pred**


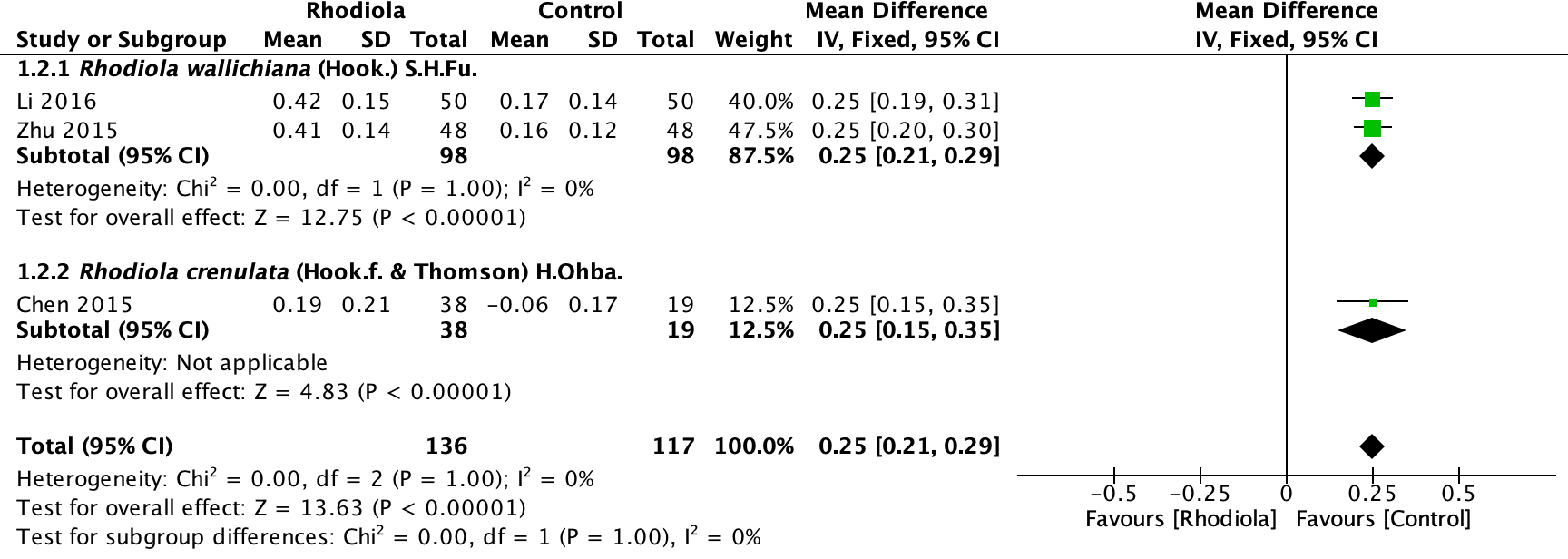


**Figure 1.2 FEV1**


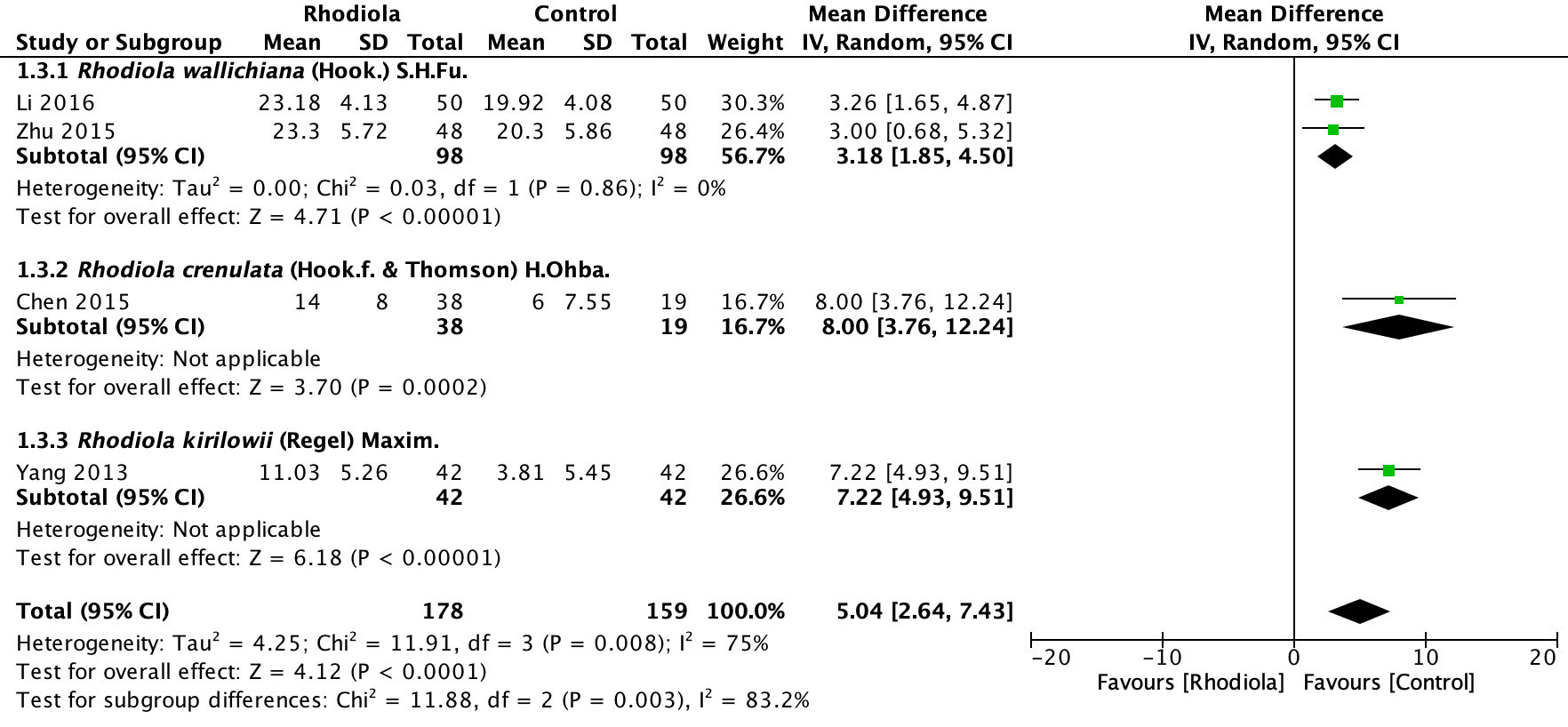


**Figure 1.3 FEV1/FVC**

**
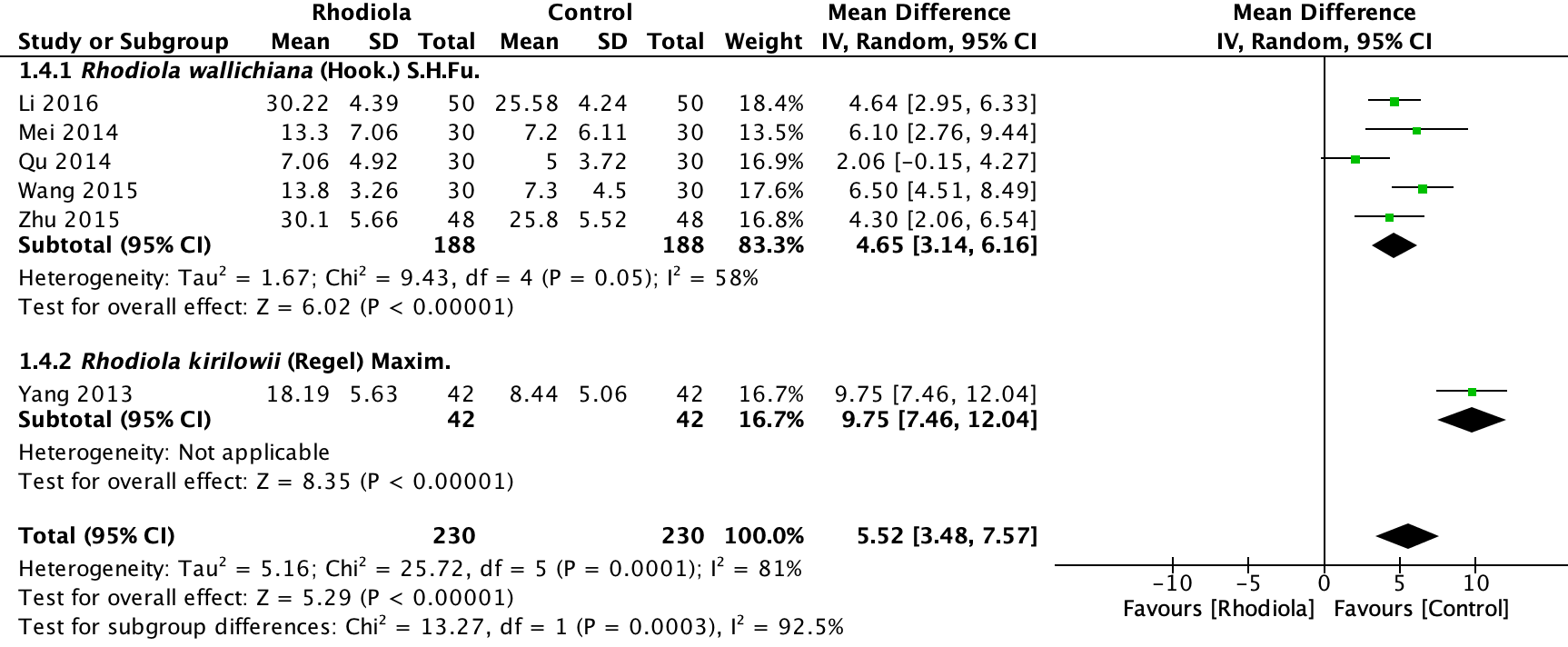
**

**Figure 1.4 PaO2**

**
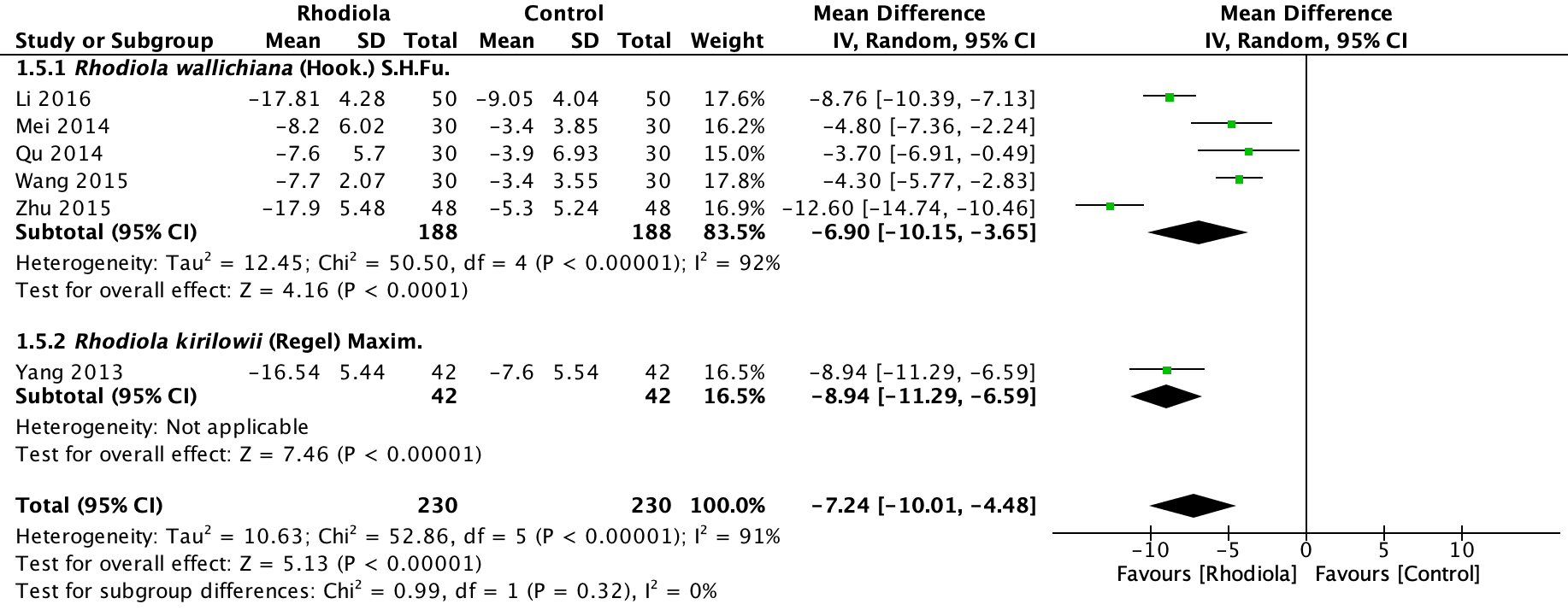
**

**Figure 1.5 PaCO2**

**
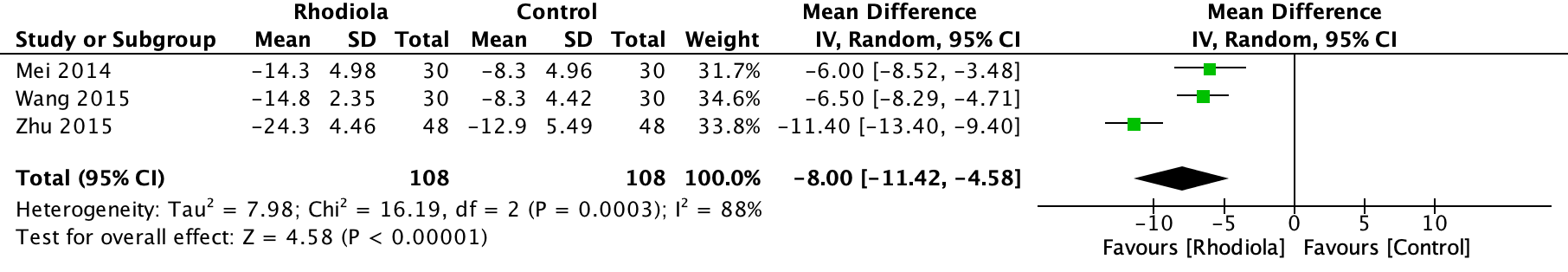
**

**Figure 1.6 SPAP**

**
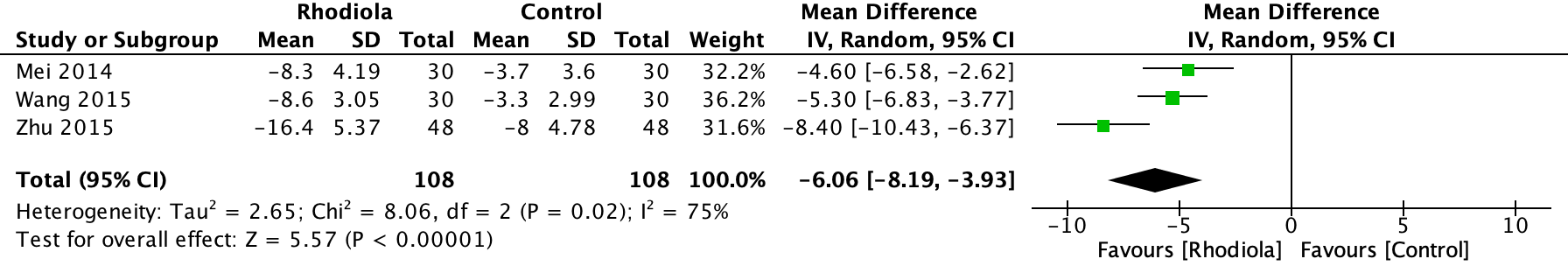
**

**Figure 1.7 dPAP**

**
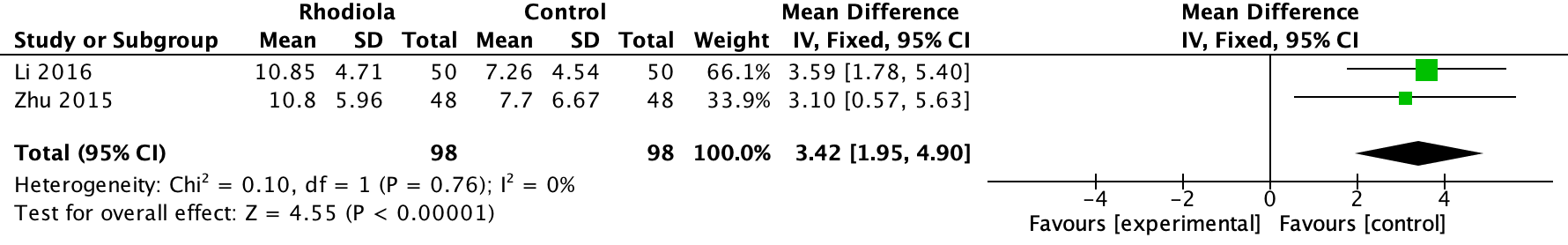
**

**Figure 1.8 SaO2**

**
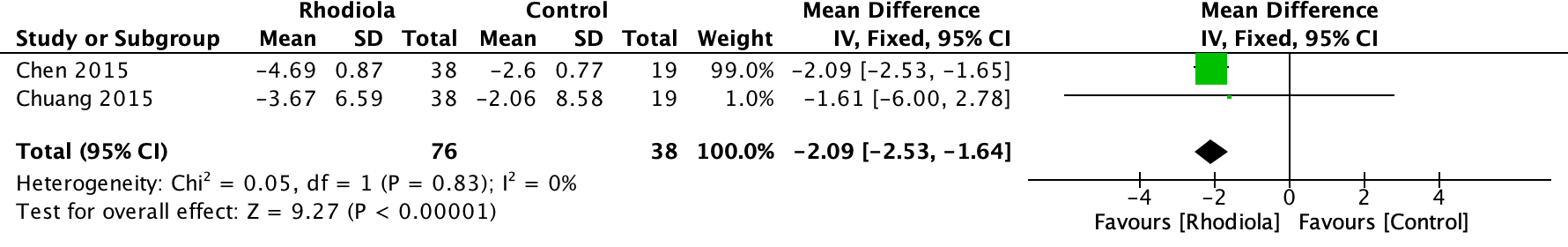
**

**Figure 1.9 CAT score**

**
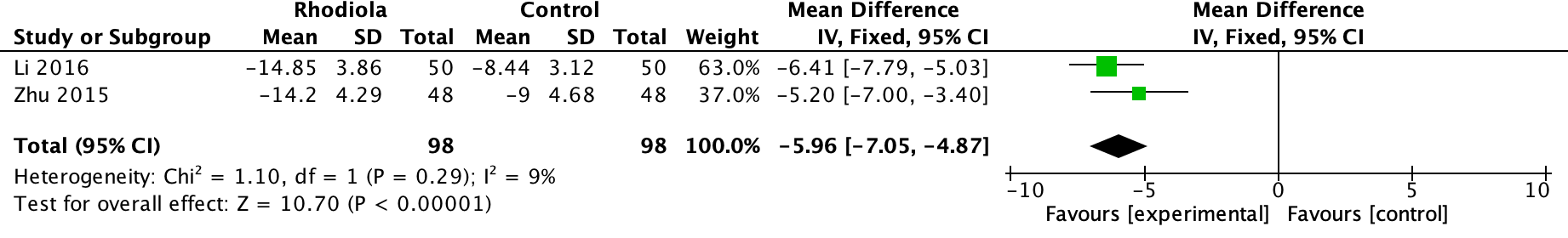
**

**Figure 1.10 CRP**

**
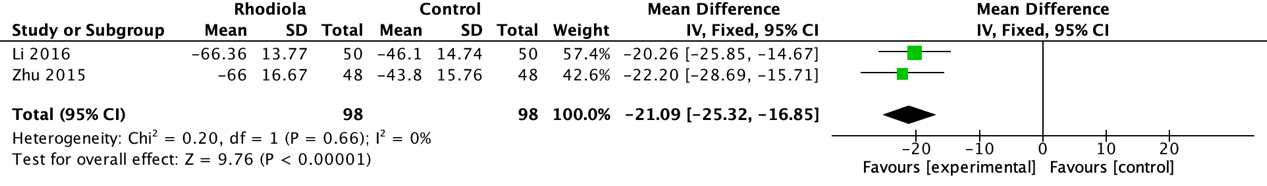
**

**Figure 1.11 NT-pro-BNP**

**
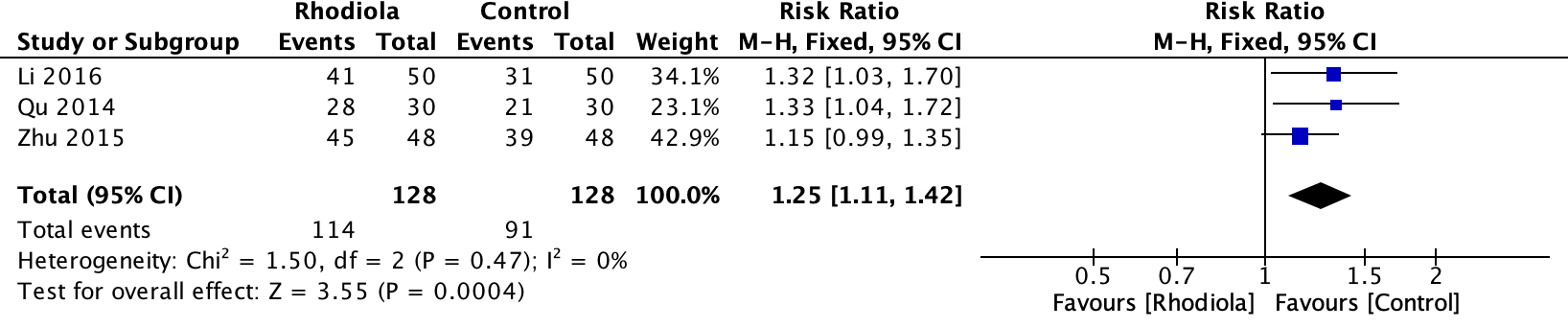
**

**Figure 1.12 Efficient Rate**

**
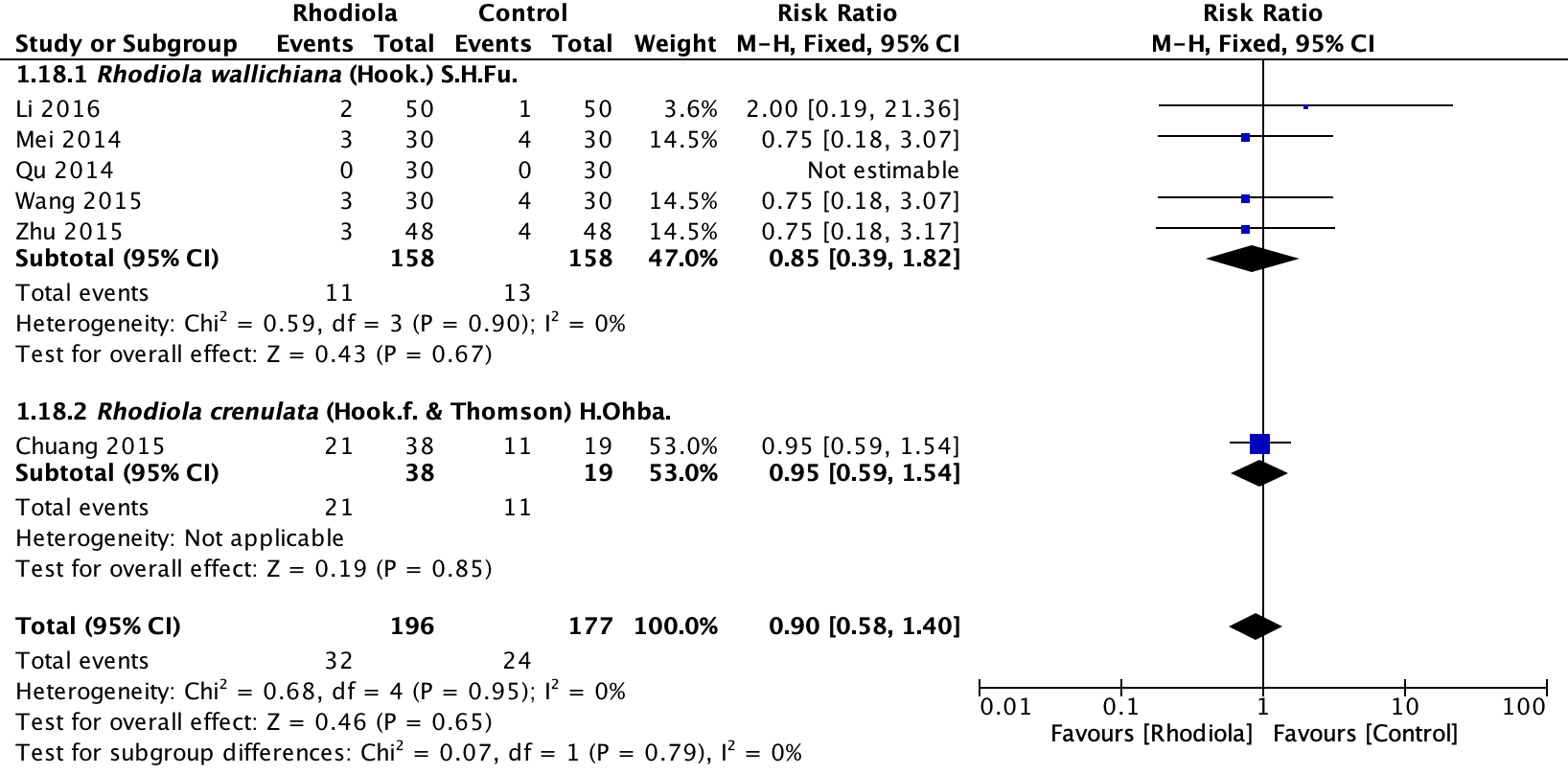
**

**Figure 1.13 Adverse Events**

**
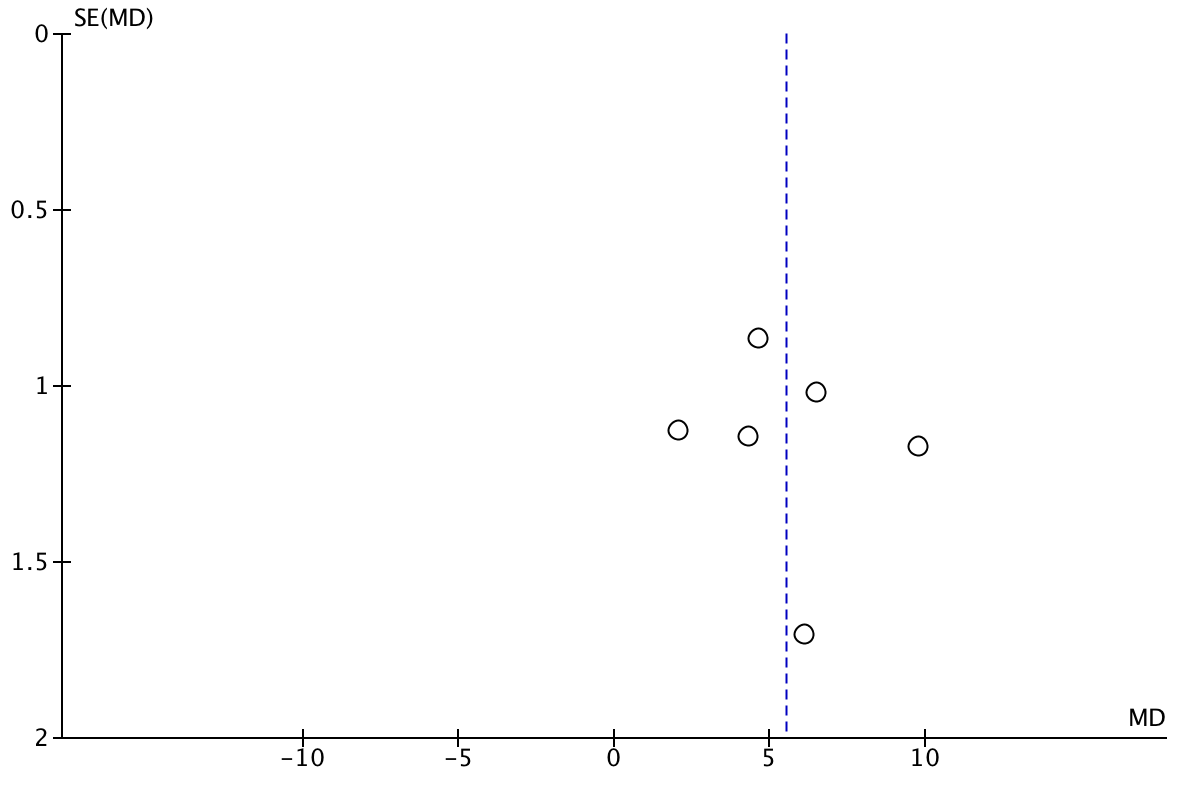
**

**Figure 1.14 Funnel Plot of PaO_2_**

**
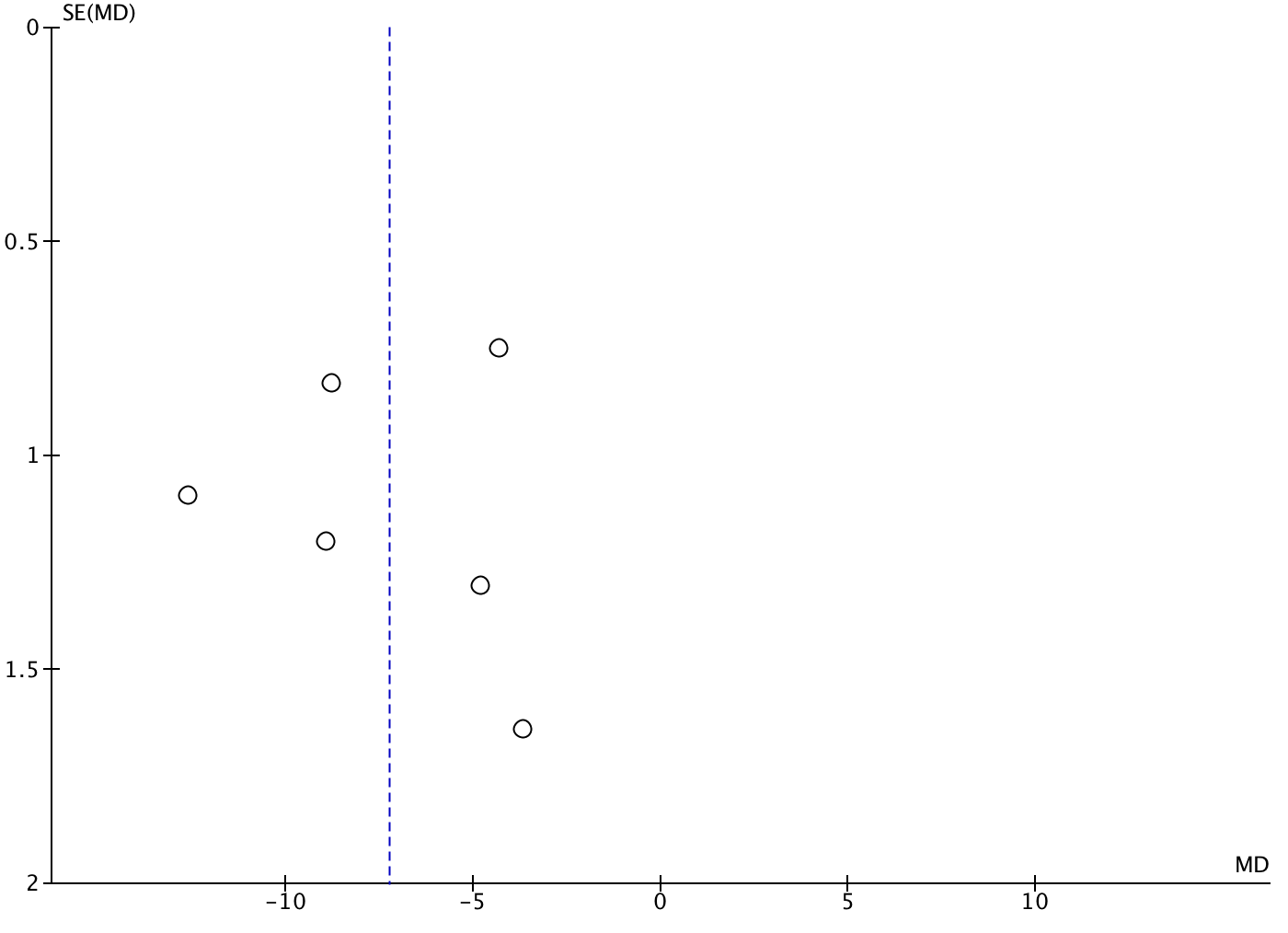
**

**Figure 1.14 Funnel Plot of PaCO_2_**

**_
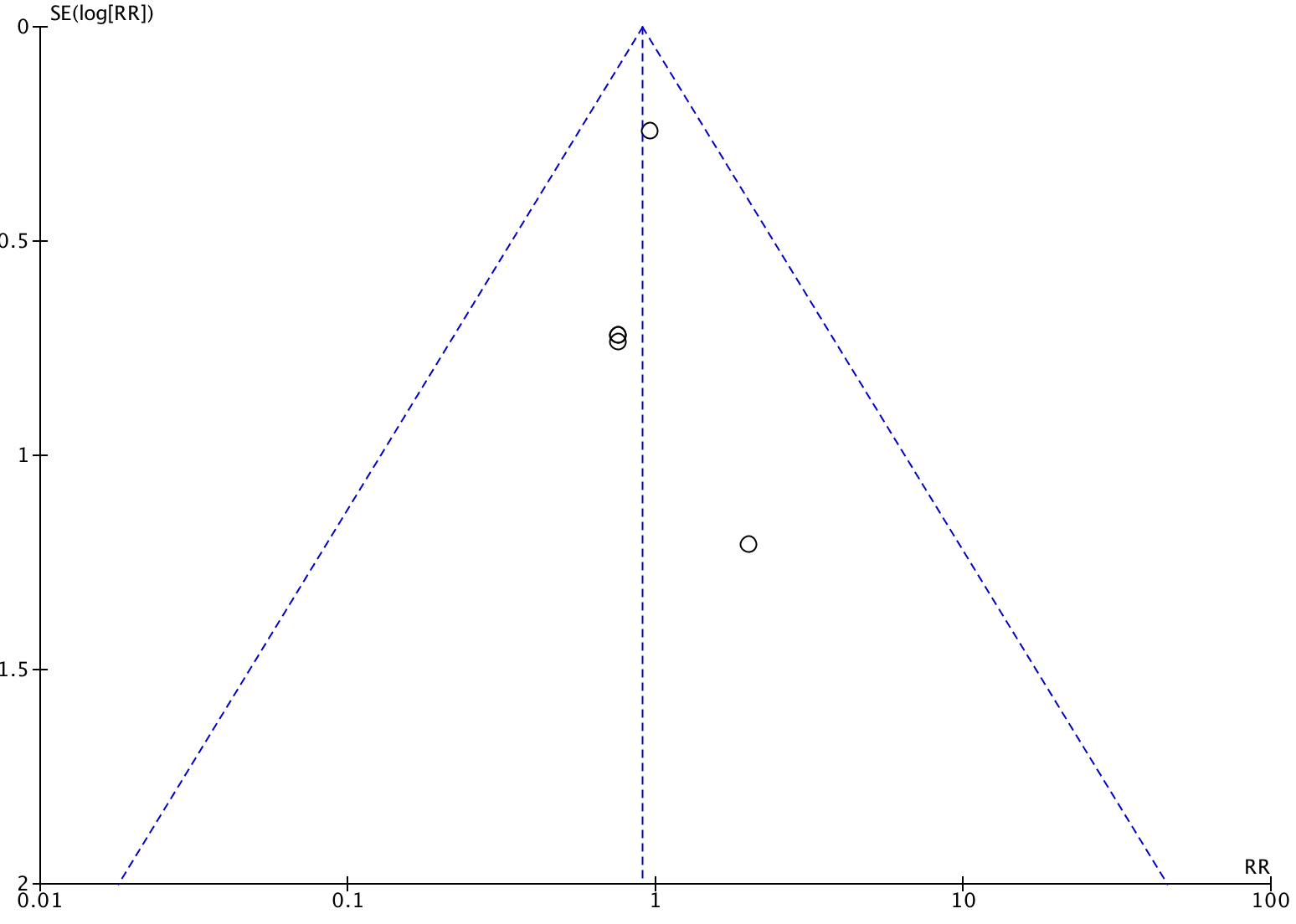
_**

**Figure 1.15 Funnel Plot of Adverse Events**

**Supplementary Figure Series 2 Forest plots (*Rhodiola kirilowii* (Regel) Maxim. vs. Ambroxol)**

**
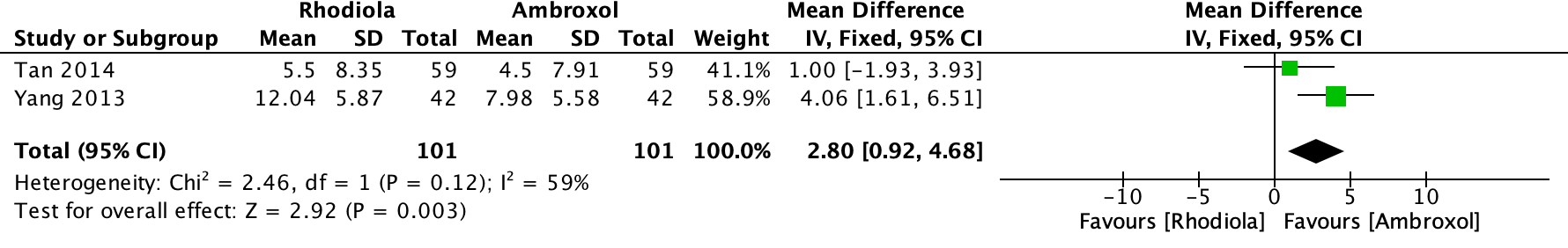
**

**Figure 2.2 FEV1%pred**

**
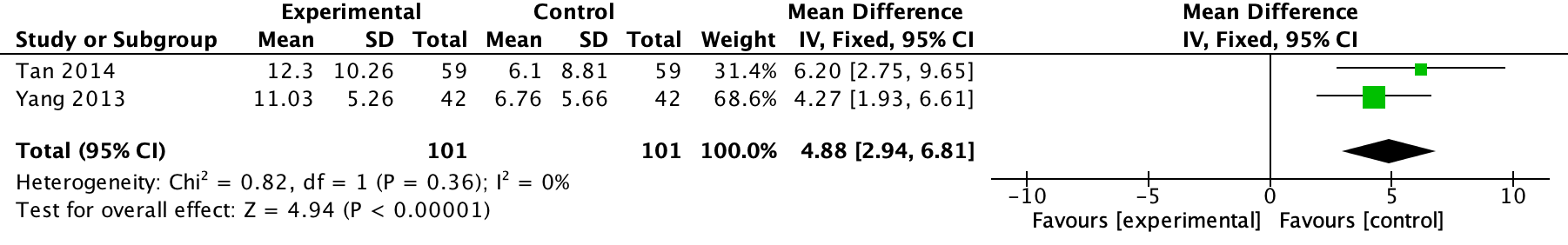
**

**Figure 2.2 FEV1**

**
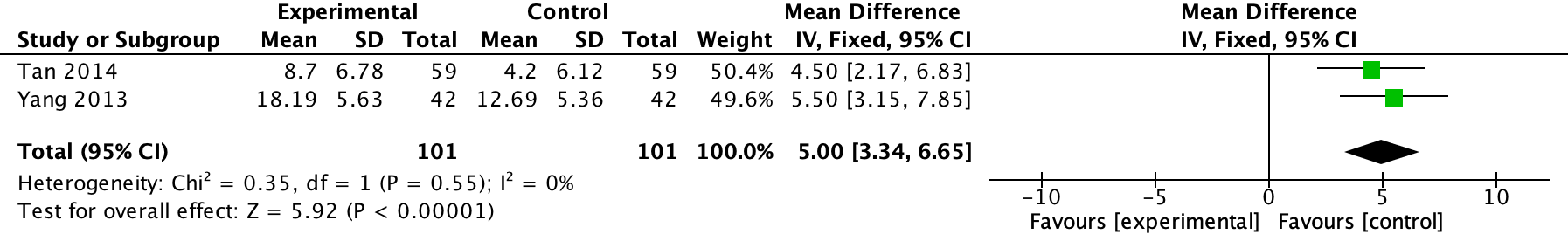
**

**Figure 2.3 PaO2**

**
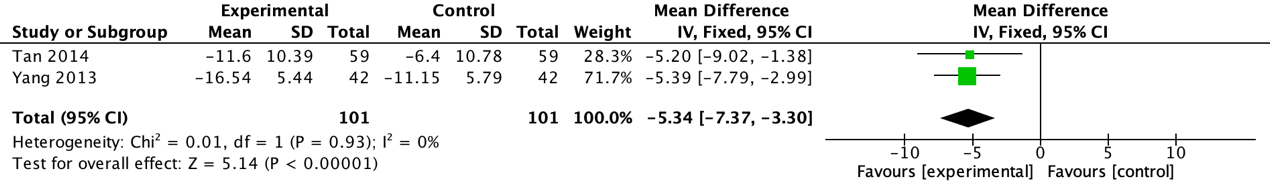
**

**Figure 2.4 PaCO2**

**
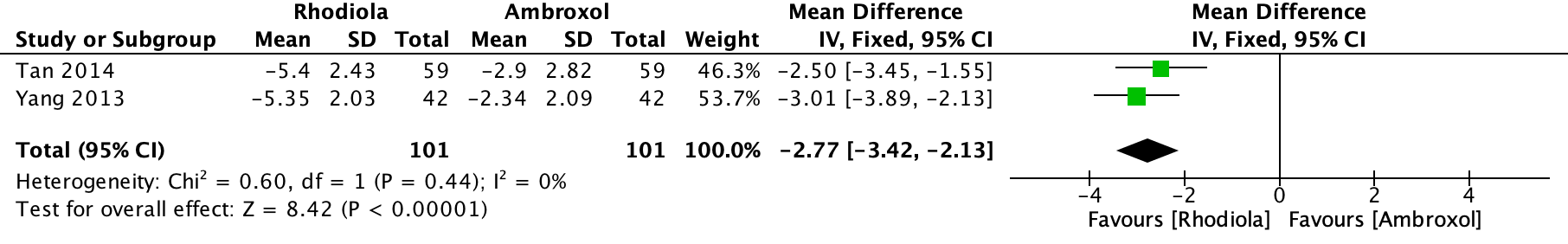
**

**Figure 2.5 8-iso PGF-2α**

**
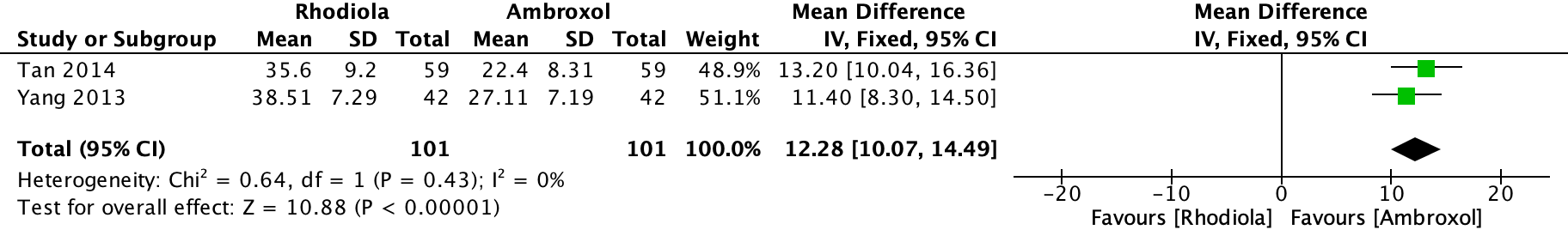
**

**Figure 2.6 SOD**

**
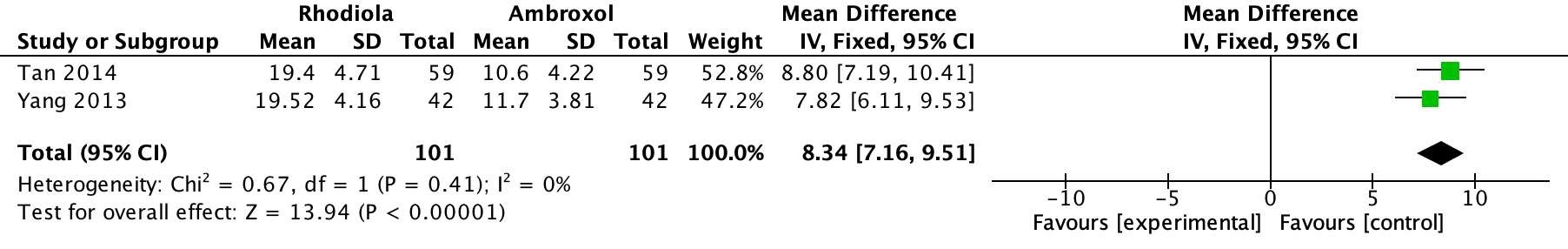
**

**Figure 2.7 GSH**

**
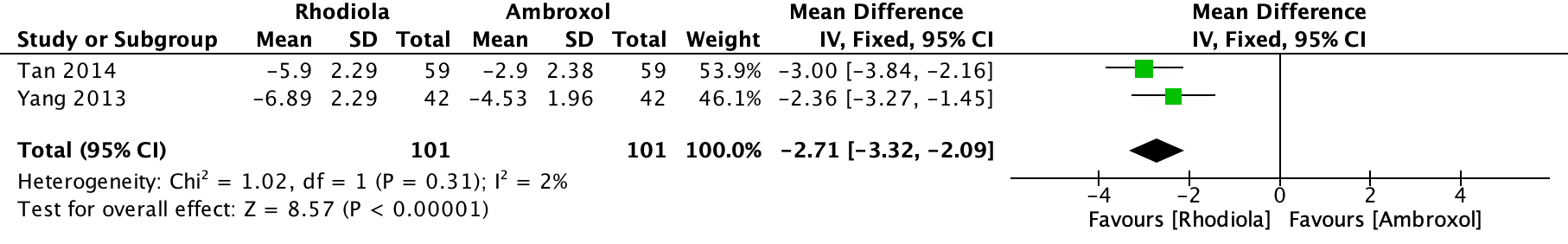
**

**Figure 2.8 MDA**

**
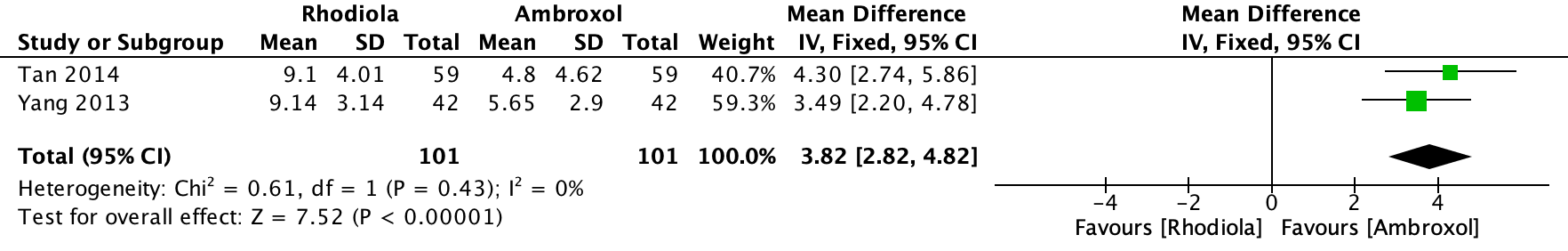
**

**Figure 2.9 T-AOC**
